# Molecular Pattern and Clinical Implications of KRAS/NRAS and BRAF Mutations in Colorectal Cancer

**DOI:** 10.3390/cimb45100491

**Published:** 2023-09-26

**Authors:** İvo Gökmen, Ebru Taştekin, Nazan Demir, Erkan Özcan, Fahri Akgül, Muhammed Bekir Hacıoğlu, Bülent Erdoğan, Sernaz Topaloğlu, İrfan Çiçin

**Affiliations:** 1 Division of Medical Oncology, Department of Internal Medicine, Trakya University School of Medicine, Edirne 22030, Turkey; theerkanozcan_57@hotmail.com (E.Ö.); fariak@doctor.com (F.A.); mbekirhacioglu@yahoo.com (M.B.H.); sernaz.uzunoglu@gmail.com (S.T.); irfancicin@hotmail.com (İ.Ç.); 2 Department of Pathology, Trakya University School of Medicine, Edirne 22030, Turkey; ebrutastekin@hotmail.com; 3 Department of Medical Oncology, Sultan I. Murat Public Hospital, Edirne 22030, Turkey; nzndr@yahoo.com.tr

**Keywords:** KRAS, NRAS, BRAF, mutation, NGS, colorectal cancer

## Abstract

The aim of our study was to evaluate the incidence of KRAS/NRAS and BRAF mutations, analyze molecular patterns, and investigate associations with clinical parameters of these mutations in CRC KRAS/NRAS and BRAF mutations analyzed by next-generation sequencing. The detection rates of these mutations and patients’ demographics were recorded and the relationship between them was evaluated using the chi-square test. KRAS mutation was detected in 332 of 694 patients, while the mutation rates in KRAS exons 2/3 and 4 were 39.6%/3.2% and 5%, respectively. The most common mutation pattern was KRAS G12D. Five atypical variants were detected: V14I in KRAS exon 2, A18D, Q22K and T50I in exon 3, and T148P in exon 4. NRAS mutation was detected in 29 (4.5%) patients. One atypical variant L80W was detected in NRAS exon 3. BRAF mutation was seen in 37 (5.3%) patients, with BRAF^V600E^ (83.8%) being the most common mutation pattern. NRAS mutation was significantly more frequent in patients > 64 years of age, BRAF mutation in women, and NRAS/BRAF mutations in right colon tumors. Grouping BRAF mutations into BRAF^V600E^ and BRAF^non-V600E^ and their analysis according to specific tumor localizations showed that all four BRAF^non-V600E^ mutations originated in the rectum. In our study, KRAS exon 2 and other RAS mutation rates were higher than in the literature, while the BRAF v.600E mutation rate was similar. NRAS and BRAF mutations were significantly more frequent in the right colon. BRAF mutation was more common in women and in the right colon.

## 1. Introduction

Globally, colorectal cancer (CRC) is the third most commonly diagnosed cancer in men and the second in women [1]. In 2020, an estimated 1.9 million new cases of CRC were reported worldwide, representing a 10% rate, leading to 935,000 deaths at a rate of 9.4% per year [2]. Approximately 20–25% of CRC cases are metastatic at the time of diagnosis and metastases may develop during the course of the disease in approximately 50% of cases [3,4]. Survival in unresectable untreated mCRC has historically been between 3 and 6 months. Before 1996, survival was 10 months with 5-fluorouracil (5-FU) monotherapy, then 20 months with 5-FU, irinotecan, or oxaliplatin-based chemotherapy (CT) used in combination or sequentially [5]; more recently, with the introduction of monoclonal antibodies (MoAbs) targeting angiogenesis and epidermal growth factor receptor (EGFR) signaling, survival has now reached approximately 30 months [6].

The development of colorectal cancer is a multi-step process involving chromosomal abnormalities, gene mutations, and epigenetic modifications [7]. A number of oncogenes play key roles in colorectal carcinogenesis [8]. The most important are oncogenic mutations of rat sarcoma virus (RAS) and b-Raf murine sarcoma viral oncogene B (BRAF), which activates the mitogen-activated protein kinase (MAPK) signaling pathway [9,10].

While the prevalence of oncogenic RAS mutations may vary regionally, they are present in approximately 50–55% of mCRC [11,12,13]. RAS mutations are negative predictive markers for treatment response to MoAbs targeting EGFR. Based on data from pivotal clinical trials, it was known that patients with mCRC whose tumors contained activating mutations in exon 2 (codon 12/13) of Kirsten-RAS (KRAS) did not benefit from cetuximab and panitumumab, or from EGFR-MoAbs combined with CT [14]. Then, retrospective analyses of the PRIME and CRYSTAL trials and new evidence from other phase II and phase III trials of EGFR-MoAbs therapies showed that other less frequent RAS mutations other than KRAS exon 2 (KRAS exon 3 (codons 59/61), exon 4 (codons 117/146), and neuroblastoma RAS viral oncogene virus (NRAS) exons 2, 3 and 4) also predicted lack of response to these MoAbs [4]. In 2015, the American Society of Clinical Oncology (ASCO) recommended an expanded RAS analysis to include other RAS mutations in all patients with mCRC who are candidates for these antibody therapies [15].

BRAF mutations are present in approximately 5–17% of CRC, with the BRAF^V600E^ mutation being the most common [16]. In mCRC, BRAF^V600E^ mutation is a negative predictive marker of treatment response to MoAbs targeting EGFR, and is associated with very poor prognosis. In first-line therapies, median survival for patients with BRAF^V600E^ (+) ranges from 9–19 months, less than half the median survival of patients with BRAF^V600E^ (−) [17]. In a first-line CALGB 80,405 study in which bevacizumab or cetuximab were combined with CT, the median survival for patients with BRAF^V600E^ (+) was 13.5 months compared to 30.6 months for patients with BRAF^V600E^ (−) [18]. In patients with mCRC, real-world data analyses also support the poor prognostic value of BRAF^V600E^ mutations (median survival of 18.2 months versus 41.1 months) [19]. Based on these data, the National Comprehensive Cancer Network (NCCN) and the European Society of Medical Oncology (ESMO) do not recommend use of cetuximab or panitumumab in patients with BRAF^V600E^ (+) mCRC [4]. However, although there is scarce data predicting the response to EGFR-targeting MoAbs for BRAF mutations other than BRAF^V600E^ in mCRC, survival is better in patients with BRAF mutations compared to those with BRAF BRAF^V600E^ (+) [20,21].

The objective of our retrospective study was to confirm KRAS/NRAS (RAS) and BRAF mutation rates in a Turkish cohort of 694 patients diagnosed with CRC using next-generation sequencing (NGS). We also analyzed KRAS/NRAS (RAS) and BRAF mutation molecular patterns, as well as their associations with clinical parameters and the literature.

## 2. Material and Method

In this study, 694 patients were included, diagnosed with histopathologically proven colorectal cancer whose molecular analysis was performed with NGS, between 2015 and 2022 at Trakya University Medical Faculty Hospital. Patients with a second malignancy at the time of diagnosis, and patients whose molecular analysis was performed by real-time PCR were not included in the study. The study was designed to be retrospective. Approval for the study was obtained from the ethics committee of Trakya University in May 2023.

NGS analysis was performed by the pathology department. Tumor tissues were evaluated regardless of the stage of disease. Here, 90.3% of the tissue samples were obtained from the primary mass, 5% from the liver, 3% from the peritoneum, and 1.6% from the lung. Isolation of genomic DNA all specimens were fixed in 10% neutral buffered formalin for 16–48 h, and then were embedded in paraffin. Tissue blocks with adequate tumor cellularity (≥30%) were assessed by an expert pathologist and then were selected to obtain genomic DNA. Two different devices and three different kits were used. Here, 52.4% of patients were analyzed with the Panel PCR kit V12 on the QIAGEN GeneReader NGS device (40724, Hilden, Germany), 28% with the Qiaseq Targeted DNA custom panel kit on the Illumina NextSeq 550 device (CA92122, San Diego, CA, USA), and 19.6% with the DNA UMI kit on the GeneReader NGS device (40724, Hilden, Germany). Sequencing results were retrieved from the hospital’s electronic information system along with their epidemiological data (age, gender, tumor location, stage, and morphology).

For statistical analysis, a chi-square test was used to observe the associations of mutated genes (KRAS/NRAS and BRAF) with clinicopathological variables (age, gender, tumor site, specific localization of the tumor in the colon, MSI phenotype). Statistical analysis was performed with the Statistical Package for the Social Science (SPSS) 23.0 software. *p* values ≤ 0.05 were considered statistically significant. The group indicated as “RAS” in the article included the whole cohort of KRAS and NRAS patients. The group referred to as “KRAS rare” defines the KRAS exon 3–4 and NRAS group, which is excluded from KRAS exon 2.

## 3. Results

Of the total 694 patients included in the study, 436 (62.8%) were male and 258 (37.2%) were female, with a male/female ratio of 1.68. Median age at diagnosis was 64.8 years (range 26–90 years) regardless of gender, with a mean age at diagnosis of 64.5 ± 10.3 years for male patients and 63.5 ± 11.9 years for female patients. The decadal distribution of colorectal cancers was 4 (0.6%) between 20–29 years, 15 (2.2%) between 30–39 years, 51 (7.3%) between 40–49 years, 141 (20.3%) between 50–59 years, 251 (36.2%) between 60–69 years, 188 (27.1%) between 70–79 years, and 44 (6.3%) between 80–90 years. The age range with the highest incidence of colorectal cancer independent of gender was 60–69.

As for tumor localization, there were 56 (8.1%) patients with cecal tumors, 68 (9.8%) with tumors of the right ascending colon, 63 (9.1%) with tumors of the transverse colon, 54 (7.8%) with tumors of the left descending colon, 159 (22.9%) with tumors of the sigmoid colon, 249 (35.9%) with tumors of the rectum, and 45 (6.5%) with tumors of the colon but no region specified. After excluding colon tumors with no specified region and rectal tumors, left-sided colon cancers (*n* = 242, 60.5%) were more predominant in our study group compared to right-sided colon cancers (*n* = 158, 39.5%). In our study, 393 patients (56.6%) had microsatellite instability (MSI)-stable, 51 (7.3%) had unstable (MSH), and the remaining 250 patients (36.0%) did not have MSI testing or had no data available.

Of all patients, there were 332 (47.8%) patients with KRAS (+), 29 (4.2%) with NRAS (+) and 37 (5.3%) with BRAF (+). KRAS/BRAF (G13D/V600E, G12V/V600E) and NRAS/BRAF (Q61H/D594G, G13R/G466V) mutations were detected in two patients each, while KRAS/NRAS (G12C/L80W) was detected in one patient. In the overall population, the incidence of RAS mutations was 52% and the incidence of other RAS mutations was 12.4%. The incidence of KRAS exon 2/3/4 mutations was 39.6%/3.2%/5%, and the most common mutation pattern was KRAS G12D (28%), followed by KRAS G13D (20.2%) and KRAS G12V (19.9%). Five atypical variants were detected: V14I in KRAS exon 2, A18D, Q22K and T50I in exon 3, and T148P in exon 4. While the incidence of NRAS exon 2/3/4 mutations was 1.3%/2.7%/0.1%, the most common mutation pattern was NRAS Q61K (37.9%) followed by NRAS Q61R (10.3%) and NRAS G13R/Q61H (6.9%). One atypical variant L80W was detected in NRAS exon 3, while codons 59 and 146 were not found. The incidence of BRAF exon 11/15 mutations was 0.4%/4.9%, with BRAF^V600E^ (83.8%) being the most common mutation pattern. The exon, codon, and molecular patterns and frequencies detected in patients with KRAS/NRAS and BRAF mutations are presented in Table 1, and the relationships of these mutations with clinical parameters are shown in Table 2.

Grouping of BRAF mutations into BRAF^V600E^ and BRAF^non-V600E^ resulted in prevalence rates of 4.5% (*n* = 31/694) and 0.9% (*n* = 6/694), respectively, in the overall population, and 83.8% (*n* = 31/37) and 16.2% (*n* = 6/37) in BRAF (+) patients. In the analysis of BRAF mutations according to colon–rectum localization, BRAF^V600E^ mutations were significantly more frequent in the colon (*p* = 0.013). When analyzed according to specific tumor localizations, BRAF^V600E^ mutations were detected in six patients in the cecum, seven in the right ascending colon, six in the transverse colon, six in the sigmoid colon, and four in the rectum, while all four BRAF^non-V600E^ mutations were of rectum origin (*p* < 0.001) (two patients had a colon origin and the specific localization was not specified). No significant correlation was found between these mutations and other clinical parameters.

## 4. Discussion

Colorectal cancer is the third most common cancer in both genders in Türkiye. According to 2020 official cancer statistics, out of 234,000 cancer cases diagnosed in 2019, 21,000 (9.1%) were CRC. Incidence and mortality rates are significantly higher in men than in women. While 26.2 new CRC cases are seen in men per 100,000 people per year, this number is 16.2 in women, and the male/female ratio is 1.61. In our study [1], 62.8% of the patients were male, 37.2% were female, and the male/female ratio was 1.68, which is consistent with the cancer statistics in Türkiye.

The mean age of diagnosis for colorectal cancer is 60 years, and it is most common in the 50–69 age range. In the United States, the proportion of new CRC cases under the age of 55 increased from 11% to 20% between 1995 and 2019 [22].

Even in young adults under the age of 39, the incidence is increasing [23]. In our study, the age at diagnosis ranged from 26 to 90 years, with a mean age of 64 years, and the proportion of patients aged 50–69 years was 56.5%, under 55 years it was 14.6%, and under 39 years it was 3%. In CRC, tumor localization is recognized as a prognostic factor and a predictive factor for treatment [24]. An analysis of colon cancers according to tumor localization revealed that there was a shift towards left-sided colon cancers, and the rate of rectal cancer increased from 27% in 1995 to 31% in 2019 [22]. Similarly, according to tumor localization, the left colon/right colon ratio was 1.5 and the rectal cancer case rate was 35.9%, according to tumor localization in our study.

In our study, the aim was to compare KRAS/NRAS and BRAF mutation rates, patterns, and their associations with clinical parameters in a Turkish cohort in the light of clinical trials, meta-analyses, and the current literature evaluating the efficacy of anti-EGFR antibodies.

First, a randomized phase II trial, OPUS, showed that there was no efficacy of cetuximab in combination therapy with first-line FOLFOX-4 in KRAS (+) patients. KRAS exon 2 mutation (codon 12/13) was found in 43% (*n* = 136/315) and BRAF mutation (BRAF V.600E) was found in 4% (*n* = 11/309) of patients. The efficacy of cetuximab was observed in patients without mutations in KRAS codon 12/13 (median survival; 18.5 versus 22.8 months) [25]. Rare RAS mutations other than KRAS exon 2 were evaluated in their archived material using the BEAMing technique after 4 years and, while rare RAS mutations were detected in 26% of patients (*n* = 31/118), the most common region of these mutations was in KRAS exon 4 (codon 117/146) with 9.3%, followed by NRAS exon 2 (codon 12/13) with 6.8%. The patients with rare RAS mutations also did not benefit from treatment with cetuximab [26].

The randomized phase III CRYSTAL study evaluated the efficacy of cetuximab in combination therapy with first-line FOLFIRI in KRAS (−) patients. KRAS codon 12/13 mutation was detected in 37% of patients (*n* = 393/1063) and BRAFV600E mutation was found in 6.6% (*n* = 70/1063). Cetuximab did not show similar efficacy in patients without KRAS codon 12/13 mutations [27]. Therefore, records from the CRYSTAL study were re-evaluated and rare RAS mutations were analyzed. Rare RAS mutations were detected in 16.1% of patients (*n* = 69/430), while KRAS codon 59/61 and codon 117/149 were found in 3.3% and 5.6% of patients, respectively, and NRAS codon 12/13, codon 59/61, and codon 117/149 were found in 3.5%, 2.8%, and 0.9% of patients, respectively. The efficacy of cetuximab in rare RAS (+) patients was not satisfactory [28].

The randomized phase III PRIME study evaluated the efficacy of panitumumab in combination therapy with FOLFOX in first-line treatment. The efficacy of panitumumab was observed in patients without KRAS exon 2 mutation (median survival; 15.5 versus 19.3 months). Mutations of KRAS codon 12/13 were found in 67% of patients, codon 61 was found in 4%, and codon 117/146 was found in 6%. Mutations of NRAS codon 12/13 and codon 61 were detected in 3% and 4% of the patients, respectively. The incidence of BRAF mutation was 8%. RAS/BRAF^V600E^ mutations had a negative predictive value for response to treatment, and this was the first clinical trial suggesting a deleterious effect of panitumumab in rare RAS (+) tumors [29]. The phase III randomized FIRE-3 study emphasizes the importance of extended RAS mutation analysis in patient selection for cetuximab treatment. Rare RAS mutations were identified in 16% of evaluable patients and overall survival was worse in patients who were carriers of these mutations (median survival 28.7 months versus 33.1 months) [30].

In our study, KRAS exon 2 mutation was observed in 39.6% of cases, while other RAS mutations were found in 12.6% of cases and BRAF^V600E^ mutation was seen in 4.9%. When compared with our study group, the incidence of RAS/BRAF^V600E^ mutations in the analyzed clinical trials was higher, except for BRAF^V600E^ in the OPUS study and KRAS exon 2 mutation rates in the CRYSTAL study.

Kafatos G et al., who evaluated the incidence of RAS/BRAF in colorectal cancer, estimated a 43.6% of incidence of RAS mutations in a meta-analysis of 4431 patients from 12 countries based on real-world data. The lowest rates were observed in Middle Eastern countries, with 33.7%, and the highest rates were observed in two European countries, Czech and Poland, with 54.1%. The incidence of BRAF mutations was estimated to be 5.8%, with the lowest rate of 2.5% in Poland and the highest rate of 14.3% in the Czech Republic [11]. While the rate of RAS mutations was higher in our study, the incidence of BRAF mutations was comparable. The highest KRAS mutation rate in the literature was reported by Rahadiani et al. [31] as 71.8% and the lowest rate was reported by Bakarman et al. as 12.5% [32]. In our study, the KRAS mutation rate was 47.2%.

Peeters M et al. aimed to determine the mutation rates of RAS/rare RAS and exons by evaluating a total of 5 retrospective clinical trials involving 3196 patients, including 3 phase III, 1 phase Ib/II, and 1 phase II. In that study, the incidence of RAS mutations was 55.9% and the incidence of other RAS mutations was 19.1%. The incidence of KRAS exon 2 mutations was 42.6%, exon 3 was 3.8%, and exon 4 was 6.2%. The incidences of NRAS exon 2, 3, and 4 mutations were 2.9%, 4.2%, and 0.3%, respectively. The incidence of BRAF mutation was reported as 8.1% [12]. In our study, KRAS exon 2, exon 3, exon 3, and exon 4 mutations were found in 39.6%, 3.2%, and 5.0% of patients, respectively, while these rates for NRAS exons 2, 3, and 4 were 1.3%, 2.7%, and 0.1%, respectively.

In a meta-analysis of 88 studies, Afolabi et al. detected the majority of KRAS mutations in codon 12 (78.2%) followed by codon 13 (21%). The rate of BRAF mutation was 5.6% [33]. While the Belgian study detected 91% of the mutations in codon 12 in KRAS (+) patients [10], this rate was only 29.3% in a study by Symvoulakis et al. in the Greek population [34]. In our study, the rate of incidence of mutations in codon 12 was 60.2%, and it 22% in codon 13 in KRAS (+) patients.

In contrast to non-small cell lung cancers, where KRAS G12C is most common, KRAS G12D and G12V are the two most common patterns in CRC [35]. In our study, KRAS G12D, G13D, and G12V were the most prevalent mutation patterns at 28%, 20.2%, and 19.9% in KRAS (+) patients and 13.4%, 9.7%, and 9.5% in the overall population, respectively, and these rates were similar to the studies of Bruera G et al. and Hamzehzadeh et al. [36,37].

Loree J et al. evaluated atypical mutation patterns in RAS (+) patients in a meta-analysis including 9485 patients from 8 cohorts. The rate of atypical RAS mutations was 1.2% (in our study, this rate was 1.5%). In terms of atypical variants, 90 were in KRAS, 39 were in NRAS, and those with an incidence ≥ 0.1% were KRAS Q22K, KRAS L19F, and KRAS D33E [38]. In our study, dive of the atypical RAS mutations were in KRAS and one was in NRAS, and the KRAS Q22K variant was 0.6% higher than the rate specified in the guidelines. These variations in the incidence of RAS/RAF mutations may be related to the study population, the percentage of tumors in the material, the sensitivity of the laboratory method, the number of codons screened for RAS mutation, or environmental factors.

In a meta-analysis by Blysma L et al. of 44 studies involving 15,981 patients from 17 European, 11 Asian, 9 US, 3 Australian, and 4 multi-country studies, the incidence of KRAS/BRAF mutations differed significantly according to tumor localization. KRAS mutations were observed in 32.4% of left-sided tumors and 41.3% of right-sided tumors (*p* = 0.017), compared to 4.3% and 16.3%, respectively, for BRAF mutations (*p* < 0.01) [39]. The incidence of NRAS did not differ significantly by localization. However, this mutation was evaluated in only three studies. In their study with a small number of patients (*n* = 151/*n* = 222), Abbasabadi M. et al. and Russo A et al. suggested that NRAS was more frequent in rectal tumors [40,41]. In our study, no significant association was found between KRAS mutation and tumor localization, whereas NRAS and BRAF mutations were significantly more frequent in the right colon.

A Brazilian cohort study including 8234 patients suggested that there is a relationship between KRAS mutations and gender, and that it is significantly higher in women than in men. There was no such relationship in NRAS [42]. In our study, there was no significant relationship between KRAS/NRAS mutations and gender, but BRAF mutations were more common in female patients. BRAF^V600E^ constitutes approximately 90% of all BRAF mutations [43], and its rate in our study was 83.8%. Compared to BRAF^non-V600E^ mutations, BRAF^non-V600E^ is detected more frequently in younger patients, males, and in left colon tumors [44]. In our study, there was no relationship between BRAF^non-V600E^ mutations, age, and gender, and all of our patients had rectal tumors.

The literature review revealed that KRAS and BRAF mutations are more common in women and right colon tumors, BRAF^non-V600E^ mutations are more common in younger patients and left colon tumors, no significant correlation could be established between NRAS mutations and clinical parameters, and there is no significant study to reveal the relationship between KRAS/NRAS and BRAF mutations and age.

### Limitations

The main limitation of this study was its retrospective design. As clinical data of the patients were not analyzed, data on the clinical presentation of the mutations could not be generated.

## 5. Conclusions

The importance of mutations and their sub-fractions in colon cancer has become better understood over the years with the development of test techniques. Thus, archival analyses were performed, and it was realized that the differences between treatment responses may also be related to atypical mutations of as yet uncertain significance. In this study, the RAS mutation rate in the Turkish cohort was revealed to be higher than in the literature, and the BRAF mutation rates were similar. As in the literature data, G12D was found to be the most common mutation pattern in patients with KRAS mutation and in the whole population. While no significant relationship was found between KRAS mutation and tumor localization, NRAS and BRAF mutations were significantly more frequent in the right colon. BRAF mutation was more common in women and tumors located in the right colon, similar to the literature. Since mutations and their clinicopathological reflections may show regional differences, we think that studies in larger series, in different regions, and different age groups, may contribute more to the literature.

## Figures and Tables

**Table 1:** KRAS, NRAS and BRAF mutation frequencies, their molecular petterns and rates.

Gene	Exon, % ^1^	Codon, % ^2^	Aa. Change, % ^3^	Nucleotide	*n*	% ^4^	% ^5^
KRAS	**2**, 82.8%	**12**, 72.7%	**p.G12D**, 46.5%	C.35G>A	93	28.0%	13.4%
	*n* = 275/332		**p.G12V**, 33%	C.35G>T	66	19.9%	9.5%
			**p.G12A**, 7.5%	C.35G>C	15	4.5%	2.2%
			**p.G12C**, 6.5%	C.34G>T	13	3.9%	1.9%
			**p.G12S**, 5%	C.34G>A	10	3.0%	1.4%
			**p.G12R**, 1.5%	C.34G>C	3	0.9%	0.4%
		**13**, 26.5%	**p.G13D**, 91.8%	C.38G>A	67	20.2%	9.7%
			**p.G13C**, 4.1%	C.37G>T	3	0.9%	0.4%
			**p.G13F**, 2.7%	C.37_38delGGinsTT	2	0.6%	0.3%
			**p.G13R**, 1.4%	C.37G>C	1	0.3%	0.1%
		**14**, 0.7%	**p.V14I**, 100%	C.40G>T	2	0.6%	0.3%
	**3**, 6.6%	**18**, 4.5%	**p.A18D**, 100%	C.53C>A	1	0.3%	0.1%
	*n* = 22/332	**22**, 18.2%	**p.Q22K**, 100%	C.64C>A	4	1.2%	0.6%
		**50**, 4.5%	**p.T50l**, 100%	C.149C>T	1	0.3%	0.1%
		**59**, 4.5%	**p.A59T**, 100%	C.175G>A	1	0.3%	0.1%
		**61**, 68.2%	**p.Q61H**, 73.3%	C.183A>C	11	3.3%	1.6%
			**p.Q61L**, 20%	C.I82A>T	3	0.9%	0.4%
			**p.Q61K**, 6.7%	C.181C>A	1	0.3%	0.1%
	**4**, 10.5%	**117**, 17.1%	**p.K117N**, 100%	C.351A>T	6	1.8%	0.9%
	*n* = 35/332	**146**, 77.1%	**p.A146T**, 81.5%	C.436G>A	22	6.6%	3.2%
			**p.A146V**, 18.5%	C.437C>T	5	1.5%	0.7%
		**148**, 5.7%	**p.T148P**, 100%	C.442A>C	2	0.6%	0.3%
NRAS	**2**, 31.0%	**12**, 66.7%	**p.G12D**, 66.7%	C.35G>A	4	13.8%	0.6%
	*n* = 9/29		**p.G12C**, 16.7%	C.34G>T	1	3.4%	0.1%
			**p.G12R**, 16.7%	C.34G>C	1	3.4%	0.1%
		**13**, 33.3%	**p.G13R**, 66.7%	C.37G>C	2	6.9%	0.3%
			**p.G13C**, 33.3%	C.37G>T	1	3.4%	0.1%
	**3**, 65.5%	**61**, 94.7%	**p.Q61K**, 61.1%	C.181C>A	11	37.9%	1.6%
	*n* = 19/29		**p.Q61R**, 16.7%	C.182A>G	3	10.3%	0.4%
			**p.Q61H**, 11.1%	C.183A>T	2	6.9%	0.3%
			**p.Q61L**, 5.6%	C.182A>T	1	3.4%	0.1%
			**p.Q61l**, 5.6%	C.181_182del	1	3.4%	0.1%
		**80**, 5.3%	**p.L80W**, 100%	C.240T>G	1	3.4%	0.1%
	**4**, 4.5%	**117**, 100%	**p.K117E**, 100%	C.349A>G	1	3.4%	0.1%
BRAF	**11**, 8.1%	**466**, 66.7%	**p.G466V**, 100%	C.1297G>T	2	5.4%	0.3%
	*n* = 3/37	**469**, 33.3%	**p.G469A**, 100%	C.1406G>C	1	2.7%	0.1%
	**15**, 91.9%	**581**, 2.9%	**p.N581I**, 100%	C.1742A>T	1	2.7%	0.1%
	*n* = 34/37	**594**, 2.9%	**p.D594G**, 100%	C.1781A>G	1	2.7%	0.1%
		**600**, 91.2%	**p.V600E**, 100%	C.1799T>A	31	83.8%	4.5%
		**601**, 2.9%	**p.K601E**, 100%	C.1801A>C	1	2.7%	0.1%

KRAS, Kirsten rat sarcoma virus; NRAS, neuroblastoma RAS viral oncogene homolog. BRAF, v-Raf murine sarcoma viral oncogene homolog B; % ^1^, proportion of exons in the mutant gene; % ^2^, proportion of codons in the mutant exon; % ^3^, proportion of molecular pattern in the mutant codon. % ^4^, proportion of molecular patterns in the mutant gene; % ^5^, proportion of molecular patterns in the overall population.

**Table 2:** KRAS, NRAS, BRAF mutations and their relationship with clinical parameters.

Characteristics	KRAS (+)	KRAS (−)	*p*	NRAS (+)	NRAS (−)	*p*	BRAF (+)	BRAF (−)	*p*
**Age**									
Mean (±SD)	63.9 ± 11.3	64.3 ± 10.5					64.9 ± 10.5	64.1 ± 10.9	
<64	146 (48.2%)	157 (51.8%)	0.8	7 (2.3%)	296 (97.7%)	**<0.05**	16	287	1.0
>64	186 (47.6%)	205 (52.4%)		22 (5.6%)	369 (94.4%)		21	370	
**Gender**									
Male	215 (49.3%)	221 (50.7%)	0.3	20 (4.6%)	416 (95.4%)	0.6	16 (3.7%)	420	**<0.05**
Female	117 (45.3%)	141 (54.7%)		9 (3.5%)	249 (96.5%)		21 (8.1%)	237	
**Localization**									
Right colon	77 (48.7%)	81 (51.3%)	0.6	1 (0.6%)	157 (99.3%)	**<0.05**	19 (12%)	139 (88%)	**<0.001**
Left colon	114 (47.1%)	128 (52.9%)		12 (4.9%)	230 (95.1%)		8 (3.3%)	234 (96.7%)	
**Localization**									
Colon	191 (47.7%)	209 (52.5%)	0.8	13 (3.2%)	387 (96.7%)	0.2	27 (6.7%)	373 (93.2%)	0.7
Rectum	133 (53.4%)	116 (46.5%)		13 (5.2%)	236 (94.7%)		8 (3.1%)	241 (96.7%)	
**MSI phenotype**									
Stable	189 (48.2%)	203 (51.8%)	0.2	16 (4.1%)	377 (95.9%)	1.0	14 (3.5%)	379 (96.4%)	**<0.05**
Unstable	18 (36.0%)	32 (64.0%)		2 (3.9%)	49 (96.1%)		11 (21.5%)	40 (78.4%)	

MSI, microsatellite instability; KRAS, Kirsten rat sarcoma virus; NRAS, neuroblastoma RAS viral oncogene homolog; BRAF, v-Raf murine sarcoma viral oncogene homolog B. *p* value < 0.05 are highlighted in bold.

## Data Availability

No new data were created or analyzed in this study. Data sharing is not applicable to this article.

## References

[1] Sung H., Ferlay J., Siegel R.L., Laversanne M., Soerjomataram I., Jemal A., Bray F. (2021). Global Cancer Statistics 2020: GLOBOCAN Estimates of Incidence and Mortality Worldwide for 36 Cancers in 185 Countries. CA Cancer J. Clin..

[2] Dekker E., Tanis P.J., Vleugels J.L.A., Kasi P.M., Wallace M.B. (2019). Colorectal cancer. Lancet.

[3] Schmoll H.J., Van Cutsem E., Stein A., Valentini V., Glimelius B., Haustermans K., Nordlinger B., van de Velde C.J., Balmana J., Regula J. (2012). ESMO Consensus Guidelines for management of patients with colon and rectal cancer. A personalized approach to clinical decision making. Ann. Oncol..

[4] Van Cutsem E., Cervantes A., Adam R., Sobrero A., van Krieken J.H., Aderka D., Aguilar E.A., Bardelli A., Benson A., Bodoky G. (2016). ESMO consensus guidelines for the management of patients with metastatic colorectal cancer. Ann. Oncol..

[5] Wolpin B.M., Mayer R.J. (2008). Systemic treatment of colorectal cancer. Gastroenterology.

[6] Chan D.L.H., Segelov E., Wong R.S., Smith A., Herbertson R.A., Li B.T., Tebbutt N., Price T., Pavlakis N. (2017). Epidermal growth factor receptor (EGFR) inhibitors for metastatic colorectal cancer. Cochrane Database Syst. Rev..

[7] Russo A., Rizzo S., Bronte G., Silvestris N., Colucci G., Gebbia N., Bazan V., Fulfaro F. (2009). The long and winding road to useful predictive factors for anti-EGFR therapy in metastatic colorectal carcinoma: The KRAS/BRAF pathway. Oncology.

[8] Markowitz S.D., Bertagnolli M.M. (2009). Molecular origins of cancer: Molecular basis of colorectal cancer. N. Engl. J. Med..

[9] Sebolt-Leopold J.S. (2008). Advances in the development of cancer therapeutics directed against the RAS-mitogen-activated protein kinase pathway. Clin. Cancer Res..

[10] De Roock W., Claes B., Bernasconi D., De Schutter J., Biesmans B., Fountzilas G., Kalogeras K.T., Kotoula V., Papamichael D., Laurent-Puig P. (2010). Effects of KRAS, BRAF, NRAS, and PIK3CA mutations on the efficacy of cetuximab plus chemotherapy in chemotherapy-refractory metastatic colorectal cancer: A retrospective consortium analysis. Lancet Oncol..

[11] Kafatos G., Niepel D., Lowe K., Jenkins-Anderson S., Westhead H., Garawin T., Traugottová Z., Bilalis A., Molnar E., Timar J. (2017). RAS mutation prevalence among patients with metastatic colorectal cancer: A meta-analysis of real-world data. Biomark. Med..

[12] Peeters M., Kafatos G., Taylor A., Gastanaga V., Oliner K., Hechmati G., Terwey J.-H., van Krieken J. (2015). Prevalence of RAS mutations and individual variation patterns among patients with metastatic colorectal cancer: A pooled analysis of randomised controlled trials. Eur. J. Cancer.

[13] Gao J., Sun Z.W., Li Y.Y., Shen L. (2012). Mutations of KRAS and BRAF in Chinese patients with colorectal carcinoma: Analyses of 966 cases. Zhonghua Bing Li Xue Za Zhi.

[14] Amado R.G., Wolf M., Peeters M., Van Cutsem E., Siena S., Freeman D.J., Juan T., Sikorski R., Suggs S., Radinsky R. (2008). Wild-type KRAS is required for panitumumab efficacy in patients with metastatic colorectal cancer. J. Clin. Oncol..

[15] Allegra C.J., Rumble R.B., Hamilton S.R., Mangu P.B., Roach N., Hantel A., Schilsky R.L. (2016). Extended RAS Gene Mutation Testing in Metastatic Colorectal Carcinoma to Predict Response to Anti-Epidermal Growth Factor Receptor Monoclonal Antibody Therapy: American Society of Clinical Oncology Provisional Clinical Opinion Update 2015. J. Clin. Oncol..

[16] Li Y., Li W. (2017). BRAF mutation is associated with poor clinicopathological outcomes in colorectal cancer: A meta-analysis. Saudi J. Gastroenterol..

[17] Modest D.P., Ricard I., Heinemann V., Hegewisch-Becker S., Schmiegel W., Porschen R., Stintzing S., Graeven U., Arnold D., von Weikersthal L.F. (2016). Outcome according to KRAS-, NRAS- and BRAF-mutation as well as KRAS mutation variants: Pooled analysis of five randomized trials in metastatic colorectal cancer by the AIO colorectal cancer study group. Ann. Oncol..

[18] Innocenti F., Ou F.-S., Qu X., Zemla T.J., Niedzwiecki D., Tam R., Mahajan S., Goldberg R.M., Bertagnolli M.M., Blanke C.D. (2019). Mutational Analysis of Patients With Colorectal Cancer in CALGB/SWOG 80405 Identifies New Roles of Microsatellite Instability and Tumor Mutational Burden for Patient Outcome. J. Clin. Oncol..

[19] Kayhanian H., Goode E., Sclafani F., Ang J.E., Gerlinger M., de Castro D.G., Shepherd S., Peckitt C., Rao S., Watkins D. (2018). Treatment and Survival Outcome of BRAF-Mutated Metastatic Colorectal Cancer: A Retrospective Matched Case-Control Study. Clin. Color. Cancer.

[20] Bellio H., Fumet J.D., Ghiringhelli F. (2021). Targeting BRAF and RAS in Colorectal Cancer. Cancers.

[21] Gong J., Cho M., Fakih M. (2016). RAS and BRAF in metastatic colorectal cancer management. J. Gastrointest. Oncol..

[22] Siegel R.L., Wagle N.S., Cercek A., Smith R.A., Jemal A. (2023). Colorectal cancer statistics, 2023. CA Cancer J. Clin..

[23] Singh K.E., Taylor T.H., Pan C.G., Stamos M.J., Zell J.A. (2014). Colorectal Cancer Incidence Among Young Adults in California. J. Adolesc. Young Adult Oncol..

[24] Nitsche U., Stögbauer F., Späth C., Haller B., Wilhelm D., Friess H., Bader F.G. (2016). Right Sided Colon Cancer as a Distinct Histopathological Subtype with Reduced Prognosis. Dig. Surg..

[25] Bokemeyer C., Bondarenko I., Hartmann J.T., de Braud F., Schuch G., Zubel A., Celik I., Schlichting M., Koralewski P. (2011). Efficacy according to biomarker status of cetuximab plus FOLFOX-4 as first-line treatment for metastatic colorectal cancer: The OPUS study. Ann. Oncol..

[26] Bokemeyer C., Köhne C.-H., Ciardiello F., Lenz H.-J., Heinemann V., Klinkhardt U., Beier F., Duecker K., van Krieken J., Tejpar S. (2015). FOLFOX4 plus cetuximab treatment and RAS mutations in colorectal cancer. Eur. J. Cancer.

[27] Van Cutsem E., Köhne C.H., Láng I., Folprecht G., Nowacki M.P., Cascinu S., Shchepotin I., Maurel J., Cunningham D., Tejpar S. (2011). Cetuximab plus irinotecan, fluorouracil, and leucovorin as first-line treatment for metastatic colorectal cancer: Updated analysis of overall survival according to tumor KRAS and BRAF mutation status. J. Clin. Oncol..

[28] Van Cutsem E., Lenz H.J., Köhne C.H., Heinemann V., Tejpar S., Melezínek I., Beier F., Stroh C., Rougier P., van Krieken J.H.J.M. (2015). Fluorouracil, leucovorin, and irinotecan plus cetuximab treatment and RAS mutations in colorectal cancer. J. Clin. Oncol..

[29] Douillard J.-Y., Siena S., Cassidy J., Tabernero J., Burkes R., Barugel M., Humblet Y., Bodoky G., Cunningham D., Jassem J. (2010). Randomized, phase III trial of panitumumab with infusional fluorouracil, leucovorin, and oxaliplatin (FOLFOX4) versus FOLFOX4 alone as first-line treatment in patients with previously untreated metastatic colorectal cancer: The PRIME study. J. Clin. Oncol..

[30] Heinemann V., von Weikersthal L.F., Decker T., Kiani A., Vehling-Kaiser U., Al-Batran S.E., Heintges T., Lerchenmüller C., Kahl C., Seipelt G. (2014). FOLFIRI plus cetuximab versus FOLFIRI plus bevacizumab as first-line treatment for patients with metastatic colorectal cancer (FIRE-3): A randomised, open-label, phase 3 trial. Lancet Oncol..

[31] Rahadiani N., Handjari D.R., Stephanie M., Krisnuhoni E. (2018). The low prevalence of colonic serrated adenocarcinoma with high KRAS mutational status at Cipto Mangunkusumo Hospital, Indonesia. Med. J. Indones..

[32] Bakarman M.A., AlGarni A.M. (2019). Colorectal cancer patients in western Saudi Arabia. Outcomes and predictors for survival over a 10-years period (2002–2014). Saudi Med. J..

[33] Afolabi H., Salleh S.M., Zakaria Z., Seng C.E., Nafil S.N.B.M., Aziz A.A.B.A., Wada Y., Irekeola A. (2022). A Systematic Review and Meta-analysis on the Occurrence of Biomarker Mutation in Colorectal Cancer among the Asian Population. BioMed Res. Int..

[34] Symvoulakis E.K., Zaravinos A., Panutsopulos D., Zoras O., Papalambros E., Sigala F., Spandidos D.A. (2007). Highly conserved sequence of exon 15 BRAF gene and KRAS codon 12 mutation among Greek patients with colorectal cancer. Int. J. Biol. Markers.

[35] Hunter J.C., Manandhar A., Carrasco M.A., Gurbani D., Gondi S., Westover K.D. (2015). Biochemical and Structural Analysis of Common Cancer-Associated KRAS Mutations. Mol. Cancer Res..

[36] Bruera G., Pepe F., Malapelle U., Pisapia P., Mas A.D., Di Giacomo D., Calvisi G., Troncone G., Ricevuto E. (2018). KRAS, NRAS and BRAF mutations detected by next generation sequencing, and differential clinical outcome in metastatic colorectal cancer (MCRC) patients treated with first line FIr-B/FOx adding bevacizumab (BEV) to triplet chemotherapy. Oncotarget.

[37] Hamzehzadeh L., Khadangi F., Ghayoor Karimiani E., Pasdar A., Kerachian M.A. (2018). Common KRAS and NRAS gene mutations in sporadic colorectal cancer in Northeastern Iranian patients. Curr. Probl. Cancer.

[38] Loree J.M., Wang Y., Syed M.A., Sorokin A.V., Coker O., Xiu J., Weinberg B.A., Vanderwalde A.M., Tesfaye A., Raymond V.M. (2021). Clinical and Functional Characterization of Atypical KRAS/NRAS Mutations in Metastatic Colorectal Cancer. Clin. Cancer Res..

[39] Bylsma L.C., Gillezeau C., Garawin T.A., Kelsh M.A., Fryzek J.P., Sangaré L., Lowe K.A. (2020). Prevalence of RAS and BRAF mutations in metastatic colorectal cancer patients by tumor sidedness: A systematic review and meta-analysis. Cancer Med..

[40] Russo A.L., Borger D.R., Szymonifka J., Ryan D.P., Wo J.Y., Blaszkowsky L.S., Kwak E.L., Allen J.N., Wadlow R.C., Zhu A.X. (2014). Mutational analysis and clinical correlation of metastatic colorectal cancer. Cancer.

[41] Mirzapoor Abbasabadi Z., Asl D.H., Rahmani B., Shahbadori R., Karami S., Peymani A., Taghizadeh S., Rad F.S. (2023). KRAS, NRAS, BRAF, and PIK3CA mutation rates, clinicopathological association, and their prognostic value in Iranian colorectal cancer patients. J. Clin. Lab. Anal..

[42] Gil Ferreira C., Aran V., Zalcberg-Renault I., Victorino A.P., Salem J.H., Bonamino M.H., Vieira F.M., Zalis M. (2014). KRAS mutations: Variable incidences in a Brazilian cohort of 8234 metastatic colorectal cancer patients. BMC Gastroenterol..

[43] Levin-Sparenberg E., Bylsma L.C., Lowe K., Sangare L., Fryzek J.P., Alexander D.D. (2020). A Systematic Literature Review and Meta-Analysis Describing the Prevalence of KRAS, NRAS, and BRAF Gene Mutations in Metastatic Colorectal Cancer. Gastroenterol. Res..

[44] Ciombor K.K., Strickler J.H., Bekaii-Saab T.S., Yaeger R. (2022). BRAF-Mutated Advanced Colorectal Cancer: A Rapidly Changing Therapeutic Landscape. J. Clin. Oncol..

